# Oligodendrocyte Progenitors Display Enhanced Proliferation and Autophagy after Space Flight

**DOI:** 10.3390/biom13020201

**Published:** 2023-01-19

**Authors:** Victoria Tran, Nicholas Carpo, Carlos Cepeda, Araceli Espinosa-Jeffrey

**Affiliations:** Department of Psychiatry, Semel Institute for Neuroscience and Human Behavior, The University of California Los Angeles, Los Angeles, CA 90095, USA

**Keywords:** space flight, oligodendrocyte progenitors, autophagy, proliferation, visual impairment intracranial pressure, intracranial hypertension, SPARC, HSP-90

## Abstract

Intracranial hypertension (ICP) and visual impairment intracranial pressure (VIIP) are some of the consequences of long-term space missions. Here we examined the behavior of oligodendrocyte progenitors (OLPs) after space flight using time-lapse microscopy. We show that most OLPs divided more than ground control (GC) counterparts did. Nonetheless, a subpopulation of OLPs flown to space presented a significant increase in autophagic cell death. Examination of the proteomic profile of the secretome of space flown OLPs (SPC-OLPs) revealed that the stress protein heat shock protein-90 beta “HSP-90β” was the 5^th^ most enriched (6.8 times) and the secreted protein acidic and rich in cysteine “SPARC” was the 7^th^ most enriched (5.2 times), with respect to ground control cells. SPARC induces endoplasmic reticulum stress, which leads to autophagy. Given the roles and importance of these two proteins in mammalian cells’ metabolism, their upregulation may hold the key to modulating cell proliferation and autophagy, in order to mitigate ICP and VIIP during and after space missions.

## 1. Introduction

It has been reported that microgravity induces intracranial hypertension (ICP) and visual impairment intracranial pressure (VIIP) in astronauts after long-duration space missions. Both ICP and VIIP remain when astronauts return to Earth. These conditions represent a risk factor and a potential limitation to long-duration space missions such as flying to the Moon or Mars. The central nervous system (CNS) is a complex system that governs voluntary and involuntary functions and behaviors. It is continually adapting to challenges from the environment to sustain its functions. We have previously reported that oligodendrocyte progenitors (OLPs), the myelin-forming cells of the CNS, proliferate more in simulated microgravity (Sim-µG) and after exposure to it [1]. We have also reported that sim-µG enhances energy metabolism in OLPs [2]. The first goal of this investigation was to ascertain if human OLPs flown to space would proliferate more after space flight. We hypothesized that OLPs would have increased proliferation while in space and that this could be contributing to ICP and VIIP in astronauts that have flown to space because the symptoms do not disappear with time back on Earth.

These findings led us to investigate (i) if space microgravity would produce similar results and (ii) how OLPs adapt to the Earth’s gravity after space flight. For this purpose, we used time-lapse imaging and analysis. Another goal was to (iii) study the secretome of OLPs exposed to space microgravity (SPC-OLPs) and compare it with ground control (GC) cells.

## 2. Methods

### 2.1. Cells and Culture System

**Figure 1:**
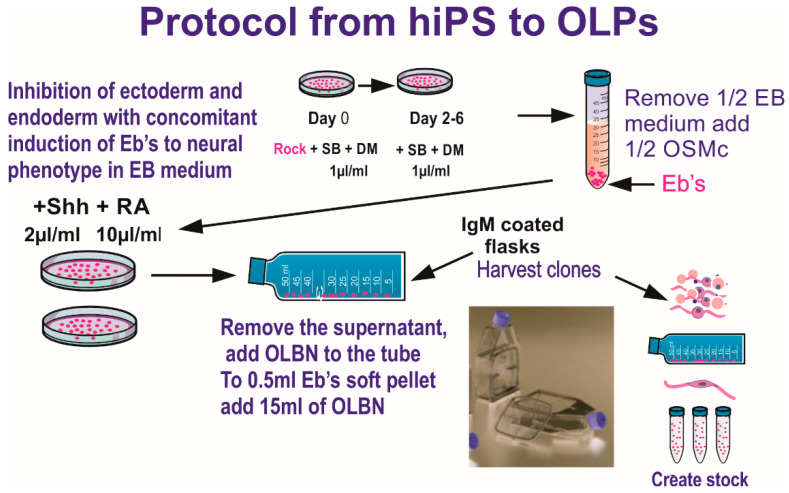
Ectoderm selection was performed by exposing hiPS to SB431542 (SB) and dorsomorphin (DM) to instruct ectoderm formation preferentially, while inhibiting endodermal and mesodermal commitment. Once specified, a stock of hiPS-derived OLPs was created for all pre-flight and post flight experiments in order to keep passage number low (18 passages maximum). Details of the procedure described here have been published [2]. This medium was designed to induce and maintain the OLP-phenotype. OLPs flew to space in OSM with N2 + B27. (Figure modified from [3]). The composition of the basal “OSM” (oligodendrocyte specification medium) is shown in Table 1.

Two years prior to the space flight, we tested several cell lines to select the most suitable for the space flight in terms of survival and hardware material compatibility, adequate seeding cell number to ensure cells would have enough nutrients for the duration of the trip, and so on. We finally selected a homogeneous population of neural stem cells, NSCs, which was obtained from human induced pluripotent stem cells, hiPS. The original hiPS cell line “CS83iCTR33-n1” (fibroblasts) was “reprogrammed” and provided to us by Cedars-Sinai Medical Center (Los Angeles CA. via a material transfer agreement. These cells were converted to “OLPs” using the protocol shown in Figure 1.

Dissolve the listed components in 500 mL of D-MEM/F12 medium (High glucose) to make OSMb (basal). To prepare OSM complete (OSMc), we added 10 µL of both N2 and B-27 with retinoic acid to OSMb. To prepare “welcoming medium” (WM), we used OSMb + IGF-1 (final concentration of 88–100 ng/mL, freshly prepared) without B-27 or N2, and allowed them to recover for six hours in the incubator prior to placing them in the imaging system.

### 2.2. Hardware and Space Flight

**Figure 2:**
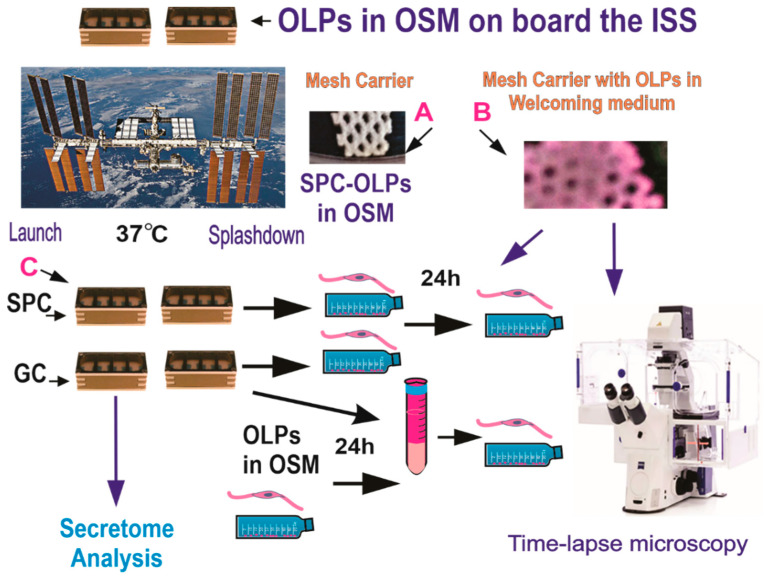
OLPs, as part of the Bioscience-4 Space Biology NASA experiment, flew to the ISS on board SpaceX-21. This experiment was launched on the 6 of December, 2020, and landed (splashed-down) on the 13 of January, 2021. Cells remained in µG at an orbit height of 254 miles. The experiment is called “passive” because it was designed to mimic the trajectory astronauts’ brains undergo during space flight (i.e., launch, stay in space, and splash down when returning to Earth) without manipulation or medium change. (**A**) Example of mesh without cells. (**B**) View of a mesh carrier populated with cells after space flight. (**C**) View of the hardware units upon return to our laboratory. Ground control cells were seeded and maintained at 37 °C in the same type of hardware and the same conditions in our laboratory, the only difference was the Earth gravity vs. space microgravity. Upon splash-down, they were transferred from Kennedy Space Center to World Carrier and brought to our laboratory (UCLA) at 37 °C.

The BioScience-4 mission launched onboard the Space-X 21 Dragon capsule on 6 December 2020. This is the first study to investigate the effects of space flight on human normal hiPS-derived OLPs. For the space flight, OLPs were seeded onto passive 8-well Petri dishes from Airbus-Kiwi (Friedrichshafen, DE), on floating mesh carriers 2 mm × 2 mm to which cells adhered firmly; this feature was necessary to ensure that cells would not detach and die during launching or while returning to Earth. OLPs were flown to the International Space Station (ISS) and installed in the STAaRS F-1 Space Technology and Advanced Research System Experiment Facility-1 (STAaRS F-1) at 37 °C. Cells remained onboard the ISS for 38 days and then returned to Earth (Figure 2).

### 2.3. Time-Lapse of SPC-OLPs Behavior after Space Flight

Upon arrival, cells were removed from the flying hardware and re-suspended in a culture medium designed to allow them to move forward in the lineage. This culture medium was made with OSMb + IGF-1 (final concentration of 88–100 ng/mL, freshly prepared) without B-27 or N2 and seeded onto T-25 flasks or flaskets with a glass bottom, at the initial seeding cell density of 0.3 × 10^6^ OLPs per flasket. OLPs were allowed to recover for six hours in the incubator prior to being placed in the imaging system. For details on the functioning of this hardware, please see information from Yuri (Available online: https://www.yurigravity.com/, accessed on 30 November 2012). We used the Zeiss Axio Observer 7 fully motorized inverted research microscope with the Zeiss Axiocam 506 monochrome camera with Zeiss ZEN software, and definite focus equipped with the full Incubation XL chamber (Zeiss) for temperature and CO_2_ control with motorized scanning stage.

### 2.4. Secretome Collection and Proteomics Analysis

**Figure 3:**
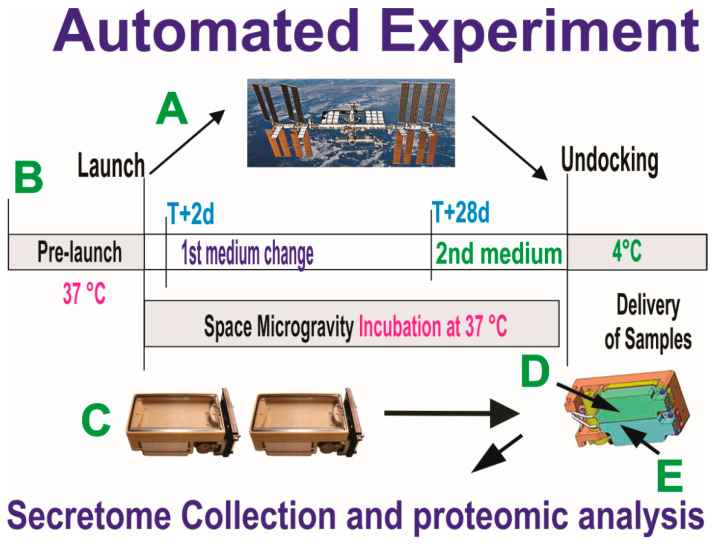
Synopsis of the automated experiments. OLPs, as part of the Bioscience-4 Space Biology NASA experiment, flew to the ISS (**A**) on board SpaceX-16, together with the passive experiments. (**B**) Timeline on when the conditioned medium (secretome) for the initial portion of the flight was removed on T+2 d after OLPs reached space. This medium was in contact with the cells for 26 days, at which time the second culture medium was recovered into the second tank just prior to unberth, in order to capture the molecules secreted solely while cells were onboarding the ISS. During ascent, descent, and space flight, cells were maintained at 37 °C. Upon arrival to our laboratory, the culture medium that fed the cells during space flight (secretome) was recovered from the hardware separately, the medium from each well was recovered separately and placed in numbered tubes with addition of proteases inhibitor cocktail, and saved frozen at −80 °C. (**C**) View of the cell chamber where mesh carriers with cells and the travel culture medium travelled. (**D**,**E**) Tanks that contained the fresh medium to be released at T+2 to start the experiment. Tank 2 (E arrow) contained the second fresh medium with which the cells came back to Earth.

For this part of the study, we chose the Automated Type IV hardware from Yuri, which allows for collecting culture medium in an automated manner. OLPs were seeded onto the mesh carriers to maximize the growth surface for the cells and to support strong adherence to it so that cells would not detach during take-off and re-entry. The experiment is summarized below (Figure 3).

### 2.5. Secretome Proteomic Profile

The conditioned medium samples (secretome) were thawed and vortexed for 20 s. The samples were spun for 30 min at 4 °C at 14,000 rpm and the supernatant was collected. For these samples, serum Albumin and IgG were removed using Thermo Scientific Albumin/IgG Removal Kit. Next, the depleted serum samples were concentrated and exchanged into 2D Lysis buffer (Sigma Millipore, St. Louis, MO, USA) (7 M urea, 2 M thiourea, 4% CHAPS, and 30 mM Tris-HCl, pH 8.5). Protein concentration was measured in all samples using the Bio-Rad protein assay method. Gel layout, Internal Standard (Cy2), OLPs_0G (Cy3), OLPs_1G (Cy5). Internal Standard was made up of an equal amount of protein from each sample. CyDye labeling: For each sample, 30 µg of protein was mixed with 1.0 μL of diluted CyDye, and kept in dark on ice for 30 min. The labeling reaction was stopped by adding 1.0 µL of 10 mM lysine to each sample, and incubating in the dark on ice for an additional 15 min. The labeled samples were then mixed. The 2X 2D Sample buffer (8 M urea, 4% CHAPS, 20 mg/mL DTT, 2% pharmalytes, and trace amount of bromophenol blue), 100 µL destreak solution, and Rehydration buffer (7 M urea, 2 M thiourea, 4% CHAPS, 20 mg/mL DTT, 1% pharmalytes and trace amount of bromophenol blue) were added to the labeling mix to make the total volume of 250 µL. We mixed well and spun before loading the labeled samples into the strip holder.

### 2.6. IEF and SDS-PAGE

After loading the labeled samples, IEF (pH3-10 Non-Linear) was run following the protocol provided by GE Healthcare. Upon finishing the IEF, the IPG strips were incubated in the freshly made equilibration buffer-1 (50 mM Tris-HCl, pH 8.8, containing 6 M urea, 30% glycerol, 2% SDS, trace amount of bromophenol blue and 10 mg/mL DTT) for 15 min with gentle shaking. Then, the strips were rinsed in the freshly made equilibration buffer-2 (50 mM Tris-HCl, pH 8.8, containing 6 M urea, 30% glycerol, 2% SDS, trace amount of bromophenol blue, and 45 mg/mL Iodoacetamide) for 10 min with gentle shaking. Next, the IPG strips were rinsed in the SDS-gel running buffer before transferring into 13.5% SDS-gels. The SDS-gels were run at 15 °C until the dye front ran out of the gels.

### 2.7. Image Scan and Data Analysis

Gel images were scanned immediately following the SDS-PAGE using Typhoon TRIO (GE Healthcare). The scanned images were then analyzed by Image Quant software (version 6.0, GE Healthcare), which is based on a scanning laser-illumination system and photomultiplier detector, followed by in-gel analysis using DeCyder software (version 5.0, GE Healthcare). The fold change of the protein expression levels was obtained from in-gel DeCyder analysis.

### 2.8. Spot Picking and Trypsin Digestion

The spots of interest were picked up by Ettan Spot Picker (Amersham BioSciences) based on the in-gel analysis and spot picking design by DeCyder software. The gel spots were washed and then digested in-gel with modified porcine trypsin protease (Trypsin Gold, Promega). The digested tryptic peptides were desalted by Zip-tip C18 (Millipore). Peptides were eluted from the Zip-tip with 0.5 ul of matrix solution (ά-cyano-4-hydroxycinnamic acid (5 mg/mL in 50% acetonitrile, 0.1% trifluoroacetic acid, 25 mM ammonium bicarbonate) and spotted on the AB SCIEX MALDI plate (Opti-TOFTM 384 Well Insert).

### 2.9. Mass Spectrometry

MALDI-TOF MS and TOF/TOF tandem MS/MS were performed on an AB SCIEX TOF/TOF™ 5800 System (AB SCIEX, Framingham, MA). MALDI-TOF mass spectra were acquired in reflectron positive ion mode, averaging 4000 laser shots per spectrum. TOF/TOF tandem MS fragmentation spectra were acquired for each sample, averaging 4000 laser shots per fragmentation spectrum on each of the 10 most abundant ions present in each sample (excluding trypsin autolytic peptides and other known background ions).

### 2.10. Database Search

Both the resulting peptide mass and the associated fragmentation spectra were submitted to the GPS Explorer workstation equipped with the MASCOT search engine (Matrix Science) to search the database of the National Center for Biotechnology Information non-redundant (NCBInr) or Swiss-Prot-database.

Searches were performed without constraining protein molecular weight or isoelectric point, with variable carbamidomethylation of cysteine and oxidation of methionine residues. One missed cleavage was also allowed in the search parameters. Candidates with either protein score C.I.% or Ion C.I.% greater than 95 were considered significant. For the analysis, we have considered only those pathways with a probability score, corrected for false discovery rate by the Benjamini–Hochberg method, *p* < 0.05.

### 2.11. Statistics

Proteomics data displayed a normal distribution (Shapiro–Wilk test) and are presented as mean ± SD. Statistical analyses for cell cycle duration and cell counts were performed using One-Way ANOVA, followed by Tukey post hoc test, in which *p* < 0.05 was defined as statistically significant.

## 3. Results

**Figure 4:**
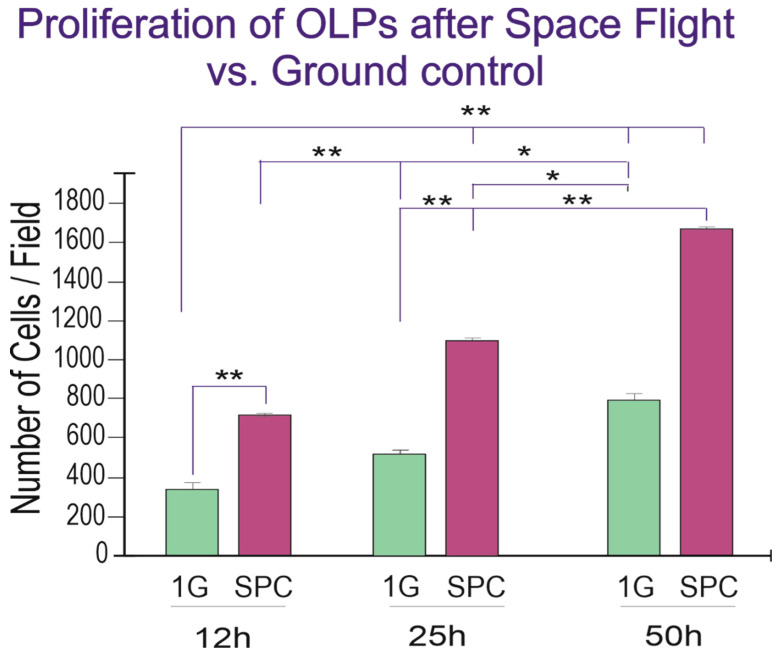
Twenty-five hours after the experiment had started there were more than twice as many OLPs and although GC cells also proliferated, there were more cells in the SPC-flown group, and these differences were significant at 12 h and 25 h, but by the 50^th^ hour, the differences were not significant anymore, as if they were tending to adapt to Earth gravity. Values are expressed as the mean ± SD of the total number of cells per field, from six different fields in three separate cultures. * *p* ˂ 0.05, ** *p* ˂ 0.01.

Upon return from the space flight on board SpX-21, OLPs were recovered from the passive flying hardware, seeded onto glass bottom flaskets and placed in the time-lapse system to ascertain cell proliferation. With time, both GC and SPC-flown OLPs increased in number, and the differences were significant across groups and at each time point studied (Figure 4).

**Figure 5:**
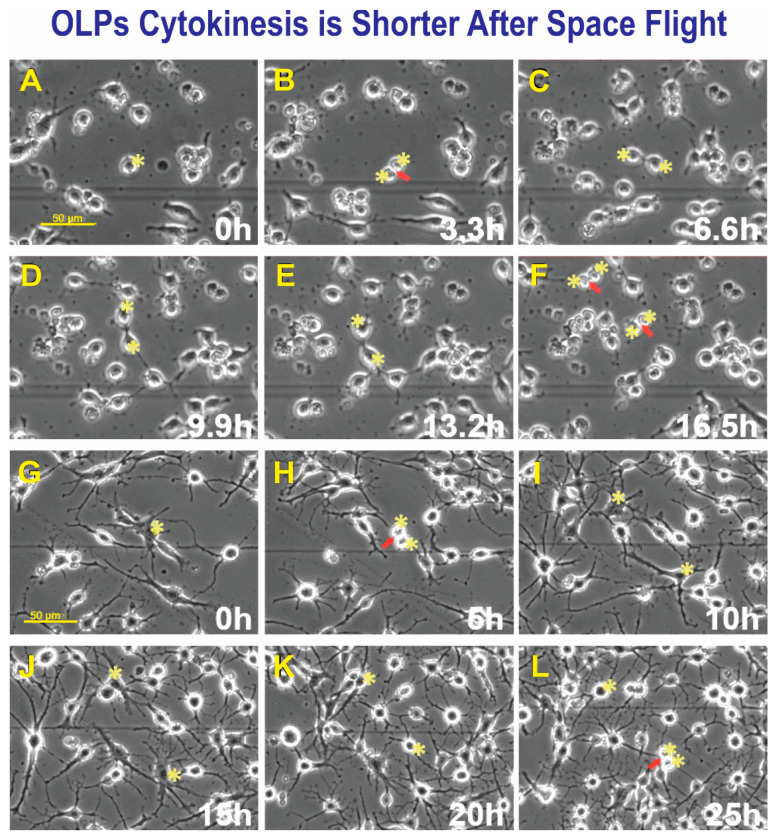
After seeding, SPC-OLPs were placed in the time-lapse microscope system with an incubator to control temperature and the CO_2_ environment. We captured phase contrast images every 10 min to visualize the changes occurring in cells after space flight and, in particular, cytokinesis. Sequential view of SPC-Flown OLPs (**A**–**F**). (**A**) Shows a cell prior to dividing, 11 h after the time-lapse acquisition started. (**B**) The cell was already dividing 3.3 h later. (**C**) Cytokinesis had been completed after 3.3 h. (**D**) Daughter OLPs—both cells had formed cell processes becoming bi-polar. (**E**) After 3.3 h, both cells looked almost the same. (**F**) After 3.3 h, both cells gave rise to two daughter cells in a total time of 6.6 h. GC-OLPs (**G**–**L**). (**G**) Ground control cells at this time point, 16.3 h after image acquisition started, OLPs were mostly bipolar or tripolar with long cell processes. (**H**) Most cells remained bipolar, but a few had developed five or six processes. (**I**) In 6 h, a cell had divided giving rise to two daughters (arrow and asterisks). (**J**) Five hours later, these cells developed multiple and robust processes (asterisks). (**K**) The appearance of the two cells was still in the same field of view and their appearance had become simpler, they bore processes but thinner and less numerous. (**L**) One of these cells divided again 20h after it had been generated (arrow). The second cell in the top right did not divide during this interval.

Space-flown OLPs had recovered well from the space flight; some were round with small, or no cell processes, and most of these cells were dividing. We also studied cytokinesis in GC-OLPs; their appearance was slightly more mature than SPC-OLPs. They bore long processes and were mostly bipolar at the beginning of the study. Nonetheless, with time, they acquired more cell processes. We found that the cell cycle in these cells was more within a normal range between 16.5 h and 25 h. Within the SPC-OLPs group, we also found a subpopulation of SPC-OLPs that divided much faster; their cytokinesis occurred within 6.6 h, at least twice. Examples of cytokinesis in space-flown OLPs and GC counterparts are shown (Figure 5A–F) and (Figure 5G–L).

### 3.1. Space-Flown OLPs Consisted of Three Distinct Sub-Populations

**Figure 6:**
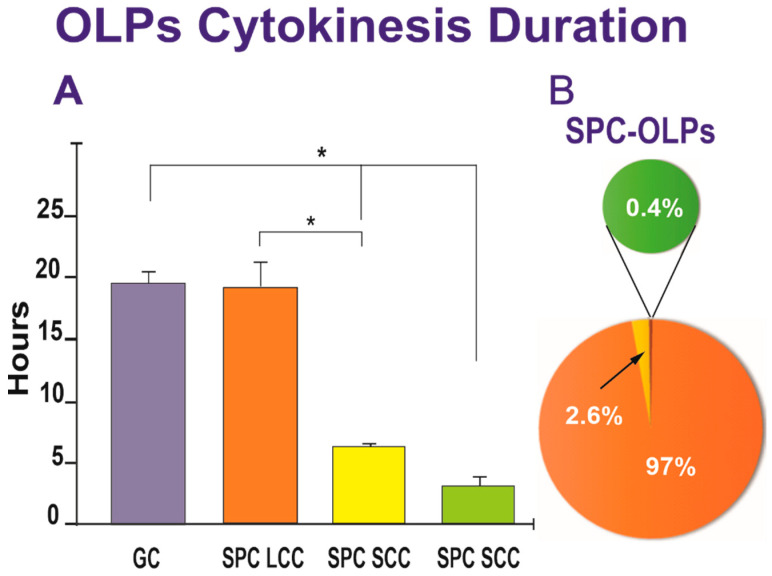
(**A**) The bar graph shows that most of the SPC-flown OLPs exhibited a long cell cycle comparable to GC-OLPs, averaging 19.43 h for SPC-OLPs and 19.76 h for GC-OLPs. The second group of SPC-OLPs divided much faster presenting cytokinesis duration of either 6.6 h (for the vast majority) or 3.3 h. One-way ANOVA showed that these differences were non-significant for the LCC group. In contrast, the differences in the short-cell cycle were significant concerning the long-cell cycle cells and the GC group. (**B**) The pie graph shows that 97% of SPC-OLPs had an average cell cycle of 19.76 h, while 2.6% displayed a shorter, and 0.4% exhibited even a much shorter cell cycle. Values are expressed as the mean ± SD of three separate cultures. * *p* ˂ 0.05.

We next examined the duration of cytokinesis in SPC-flown and GC-OLPs. Once a cell was identified, it was then studied using reverse-time tracking and verified forward. The cell cycle time was defined as the interval between when a cell was first generated by cytokinesis and when that cell subsequently divided, giving birth to two daughter cells. Quantitative data for both groups, GC-OLPs and SPC-OLPs, showed that GC-OLPs’ cytokinesis ranged between 13.66 h and 20.6 h, averaging 19.76 h. In the case of SPC-OLPs, the vast majority of SPC-flown OLPs had a cell cycle ranging between 14.33 h and 43.3 h with an average of 19.43 h; these differences were not significant (Figure 6A). Thus, the initial study on the duration of the cell cycle from space-flown OLPs appeared to show that the cells had not proliferated significantly faster after space flight when compared to GC-OLPs. Nonetheless, a detailed analysis demonstrated the existence of three subpopulations of OLPs, one exhibiting a long cell cycle (LCC) averaging 19.43 h and a second and third, with smaller OLPs groups exhibiting shorter cell cycles (SCC) with a duration of either 6.6 h or 3.3 h. (Figure 6).

### 3.2. Space Flight Upregulates Autophagy in OLPs

**Figure 7:**
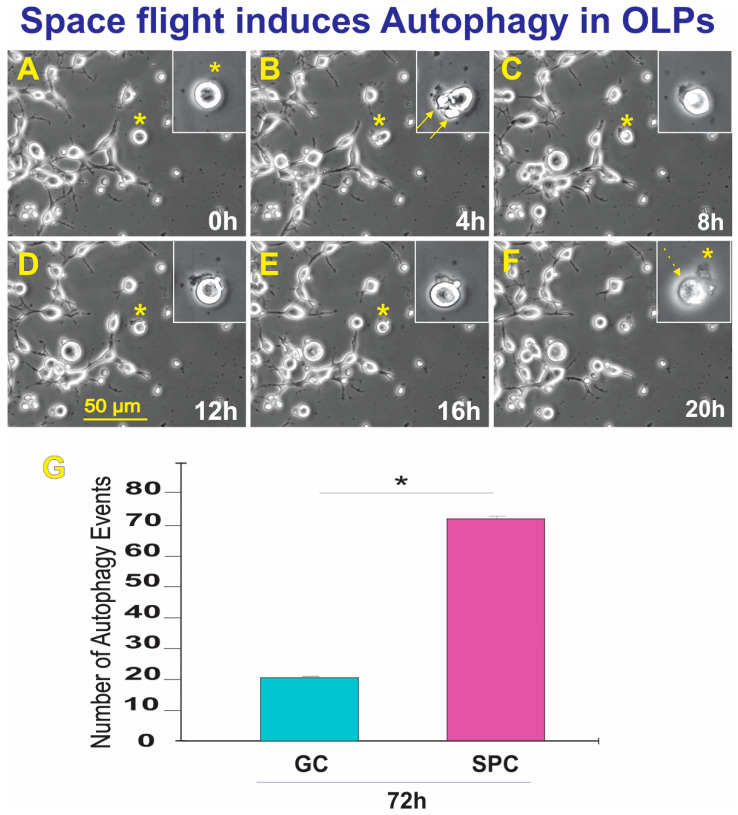
The field was examined to identify OLPs undergoing autophagy. Once a cell was identified, it was then studied using reverse-time tracking. (**A**) View of the round cell being studied 0 = 13.25 h after the start of the time-lapse capture. (**B**) Morphological changes occur where bleb-like structures are seen (arrows). The inset shows that the cell has left flat membrane-like material outside. (**C**) 8 h after, the cell changed morphology, looking like one round structure. (**D**) During the lapse of 4 h, the cell had deposited some of its contents onto the extracellular space. (**E**) Four hours later, the cell appeared to have internalized part of the expelled material, (**F**) The cell then contracted until there was just a small pocket-like structure and an empty sac-like structure. The event was completed within the lapse of 20 h—also see supplemental time lapse (TL) TL-1. (**G**) This bar graph shows the incidence of autophagic cell death in GC and SPC-flown OLPs three weeks after returning to Earth. The average total number of cells per field displaying autophagy in the GC group was 22 OLPs. In contrast, in SPC-OLPs cultures, 72 cells exhibited such behavior. Statistical significance was assessed by one-way ANOVA in which *p* < 0.05 was defined as statistically significant (*) *p* < 0.01.

Upon return, OLPs were transferred from Kennedy Space Center to UCLA. OLPs were recovered from each well separately and seeded onto separate poly-d-lysine (PdL) coated flaskets using OSM medium supplemented with IGF-1. We observed that the vast majority of the cells proliferated and migrated as expected. Six hours after being seeded, OLPs were placed in the time-lapse microscope system with an incubator to control temperature and the CO_2_ environment. We captured phase contrast images every 10 min to visualize cell changes after space flight. These cells had recovered well from the space flight, were highly migratory, and the vast majority presented bipolar or tripolar morphology. Nonetheless, some were round and adhered to the substratum, but without cell processes. In addition, we also found that some of these OLPs flown to space displayed autophagy; the number of autophagy instances is shown in Figure 7. Thus, although only a small percentage (0.12% of GC vs. 0.22% of SPC) of cells displayed autophagy, significantly more of these events were observed after space flight.

### 3.3. Secretome Proteomics

The culture medium that fed the cells during space flight was recovered from each tank separately. This medium is commonly known as the conditioned medium. For the purpose of this paper, we refer to it as “secretome”. Examination of the secretome of SPC-flown OLPs, using mass spectrometry-based quantitative proteomics, revealed that heat shock protein 90 alpha/beta (HSP-90αβ) was the fifth most abundant, followed by SPARC seventh, and HSP90β eighth most abundant, and they were significantly increased in the secretome produced while OLPs were in space. Their enrichment, with respect to Earth-grown OLPs, is shown (Table 2). Other proteins within the first seven most enriched were Glyoxalase Domain Containing 4, GC protein, prolyl 4-hydroxylase subunit beta, and Homeobox C11; these are to be discussed in separate papers currently in preparation.

## 4. Discussion

Gravity, the force that impacts our development, daily lives, and metabolic processes, is an integral part of our life, physiology, and cognition. The absence of gravity or its variation would consequently alter the development and function of all living organisms and systems. The space era has brought knowledge on the response of biological systems to microgravity. Weightlessness has been shown to cause cell damage and impair the cell cycle in various biological systems, including transiently in NSCs [4,5]. We and others [1,2,6,7] have started to unravel how CNS biological processes occur in µG. We have previously shown that the cell cycle is shortened and that more OLPs are produced in sim-µG [1]. We have also shown that OLPs exhibited an elevated energetic state and enhanced lipid secretion by human OLPs after 72 h in sim-µG [2]. Therefore, the absence of gravity provides us with a unique opportunity to gain new insights into the developmental and functional aspects of the CNS. With this in mind, we sought to determine how OLPs would behave upon exposure to space-µG.

### 4.1. OLPs Cytokinesis after Space Flight

Cell cycle regulation and functional maturation in oligodendroglia (OLs) are two critical functions that, when in balance, ensure normal CNS development, as well as healthy OLs and myelin throughout the life of the organism. Upon lineage commitment, OLPs are generated mainly through symmetric self-renewing divisions [8]. OLPs’ cell cycle length in embryonic (E) life varies, and its frequency increases from 6 h (h) to 13 h, and subsequently, it extends up to 22 h in embryonic life E-17 [9]. Debate still exists on whether the deceleration of OLPs’ cell cycle is an inherent property of OLPs, whether extracellular signals determine it, and whether the microenvironment is conditioned by themselves and/or the presence of added mitogens and other trophic factors, including neuronal signaling [10]. We believe that both aspects act in concert to support OLP maturation towards myelinating oligodendrocytes. Initially, space-flown OLPs appeared to have not proliferated significantly more after space flight when compared to GC-OLPs. Nonetheless, a more exhaustive analysis demonstrated the existence of three subpopulations of OLPs, one exhibiting a long cell cycle similar in length to the GC cells, and two exhibiting shorter cell cycles of either 6.6 h or 3.3 h. Thus, compared to reports indicating that the perinatal OLPs cell cycle averages 16 h [11], our SPC-OLPs appeared to cover both the dividing pace of the early embryonic and perinatal OLPs cell cycle. Yet, we have no explanation for the even shorter cell cycle. We and others have described the well-defined developmental stages of OLs, from OLPs to mature myelinating OLs [12]. Each stage has “stage-specific” markers, which are molecules made by these cells as they progress along their lineage. Although our resources and cell numbers obtained after space flight did not allow for epigenetic analyses, the proteomic analysis of SPC-flown secretome was very instrumental: it revealed that while in space, OLPs secreted 3.5 times more transferrin than GC-OLPs, indicating that they were at an early OLP stage while in space [3]. Moreover, their proliferative characteristics acquired while in space were preserved upon return to Earth, as if they remembered having been in µG [3].

The global proteomics analysis is still an ongoing project. Proteins within the first seven most enriched were Glyoxalase Domain Containing 4, GC protein, prolyl 4-hydroxylase subunit beta, and Homeobox C11. The potential relevance of these proteins will be discussed in separate papers currently in preparation. Nonetheless, we considered it of utmost importance to report the abundance of HSP90αβ, HSP90β, and SPARC because these proteins relate directly to the behavior of space-flown OLPs, as well as to autophagy. These experiments were designed to ascertain what molecules were produced by OLPs while in space. We were very successful because we discovered that OLPs are the cell population we should focus our efforts on to address ICP and VIIP. A cluster of molecules involved in essential roles in mammalian cellular homeostasis are the HSP90 family, and co-chaperones are the most important elements for the regulation of cytosolic HSP90. We have recently reported, using immunofluorescence, that OLPs flown to space expressed HSP-90β [3]. Proteomic analysis of the secretome demonstrated upregulation of this gene product. On Earth, the cytosolic form of HSP90 supports cell survival and controls the cell cycle, among other roles [13]. Several types of stress including reactive oxygen species, irradiation, and tissue injury result in secretion of HSP90α [14], and secreted HSPαß is beneficial because it promotes cell motility for wound healing [15]. Yet, some secreted forms have been reported to contribute to the development and progression of serious cancers and neurodegenerative diseases [13]. Therefore, ascertaining the enhanced presence of HSP90αβ and HSPβ in SPC-OLPs opens the possibility of using it as a target molecule to modulate cell proliferation in astronauts’ brains. Nonetheless, extensive research is needed to understand how these molecules interact in the entire brain, as opposed to one cell type in culture. Understanding the regulation and function of the HSP90 family members in microgravity is of the essence to harness specific countermeasures towards prevention and treatment of IP in astronauts.

### 4.2. Enhanced Autophagy in SPC-OLPs

Degradation and recycling of dysfunctional proteins or whole organelles is a lysosome-mediated mechanism known as autophagy. This process ensures homeostasis in the CNS and other tissues [16,17] and it may lead to either cell survival or cell death, depending on the type and extent of the insult or cellular stress [18]. A protein acidic and rich in cysteine, “SPARC”, is involved in autophagy, and it is a matrix-associated protein. It is required for calcification of collagen in bone too, but it is also involved in extracellular matrix synthesis and cell morphological changes. In addition, SPARC has been associated with tumor suppression. Nonetheless, it has also been correlated with metastasis and tumorigenesis. Its capacity to induce a tumorigenic phenotype has been attributed to the modulation of signaling for integrins and growth factors influencing malignant progression, including extracellular matrix remodeling, angiogenesis, immune modulation, and metastasis. In the CNS, SPARC upregulates glioma matrix by promoting collagen fibrillogenesis [19]. On the other hand, SPARC induces endoplasmic reticulum stress, which leads to autophagy [20]. Recent studies have provided evidence suggesting that simulated µG, using random positioning machines (RPMs), activate autophagy in RAW264.7 pre-osteoclast cells and seminomal cells [21,22]. Moreover, autophagy protects against µG-induced ER stress in HUVEC cells [23]. In the present study, SPARC was the seventh most expressed protein in the secretome from SPC-OLPs. When compared with the secretome from ground control OLPs, the level of SPARC contained in the secretome was 5.1 times higher. The pleiotropic effects of SPARC reflect the complexity of its interactions depending on the cell type. It plays roles in pro and anti-proliferation; it elicits endothelial reticulum (ER) stress that leads to autophagy-mediated apoptosis; it is a cell morphogen and can act as an oncogene, as well as tumor suppressor.

Thus, understanding the effects of space flight on OLPs and how to modulate such effects are of the essence, as astronauts embark on more extended duration space travel such as to the Moon and Mars. Both of our OLPs, ground control and space-flown, remained unattended for 45 days, which could be considered a stressful situation, and which would suggest that autophagy occurred out of starvation. Nonetheless, while we found autophagic behavior in GC-OLPs, these events were significantly increased in SPC-OLPs. In addition, the time-lapse study was performed on cells that were fed with fresh medium prior to the start of imaging. Thus, space flight enhanced the autophagic properties of OLPs, which indicates that cells remembered having been transiently in microgravity and that despite being back to terrestrial gravity, these physiological changes while in space persisted. Epigenetic analysis to better understand these “memory” mechanisms is of the essence. Nonetheless, increased proliferation in SPC-OLPs and SPARC upregulation explain how nature may be using this mechanism to compensate for increased proliferation by increasing autophagy in OLPs exposed to space microgravity to maintain healthy cell numbers. Although our results do not demonstrate the occurrence of this phenomenon in the astronauts’ brains, it is plausible because intracranial hypertension does not subside after returning to Earth.

### 4.3. Benefits Expected for Astronauts Living and Working in Microgravity

We have continued to unravel the effects of space flight on OLPs. The data presented here are examples of changes produced by microgravity on OLPs. Although these changes appear not to be deleterious to the vast majority of OLPs, they are very significant and long-lasting. Both OLPs proliferation and autophagy were enhanced by microgravity. Both mechanisms appear to exclude each other; the more cells there are in the brain, the more autophagy increases, resulting in benefits to astronauts that continue to live long lives upon returning to Earth. Moreover, to live on the Moon or Mars, gravity might mitigate the two phenomena to some extent, but they might persist while living in space. More research is needed to ascertain if both phenomena eventually subside as a function of time on Earth. An epigenetic study to understand the regulation of specific genes involved in both proliferation and autophagy will provide crucial information to modulate both phenomena in astronauts’ brains, and alleviate intracranial hypertension during and after space flight.

### 4.4. Benefits Expected for Humanity on Earth

Originally, our long-term goal was and still is to produce healthy, transplantable, and functional OLPs in adequate numbers to be used for cell replacement therapies to treat myelin deficiencies and cerebral palsy on Earth. The use of the microgravity approach for a short term exposure i.e., 24 h to 72 h will be beneficial in that it would expedite the production of healthy OLPs to address both developmental disabilities and degenerative neural disorders, such as in the premature neonate or patients with other leukodystrophies and neurodegenerative disorders, such as MS for which currently there are no cures. Nonetheless, one must be cautious when using microgravity to generate large cell populations as other behaviors, such as a shortening of the cell cycle, may arise.

## Figures and Tables

**Table 1:** Preparation of OSM basal.

Reagents	Measurements
Insulin	5 mg
Transferrin	50 mg
Putrescine	16.1 mg
Sodium Bicarbonate	2.2 g
D(+) galactose	4.6 g
Kanamycin	8 µg
* Sodium selenite	0.8 mg/mL

* Sodium selenite. Prepare a “stock solution” 0.8 mg/mL in PBS. Add 4 µL of the stock solution for 500 mL medium. Adjust the pH to 7.4 (after filtering).

**Table 2:** Accession information showing the abundance of HSP90αβ, SPARC, HSP90β1, and HSPA8 proteins in SPC-OLPs’ secretome that were significantly up-regulated with respect to GC-OLPs’ secretome.

SPC-OLPs > GC-OLPs			
Relativity	Gene	Accession No.	Top Ranked Protein Name [Species]
6.8	HSP90AB1	HS90B_HUMAN	Heat shock protein HSP 90-beta OS = Homo sapiens OX = 9606 GN = HSP90AB1 PE = 1 SV = 4
5.2	SPARC	SPRC_HUMAN	SPARC OS = Homo sapiens OX = 9606 GN = SPARC PE = 1 SV = 1
4.7	HSP90B1	ENPL_HUMAN	Endoplasmin OS = Homo sapiens OX = 9606 GN = HSP90B1 PE = 1 SV = 1

## Data Availability

All data obtained from this study has been included in the manuscript.

## Abbreviations

ICP	Intracranial hypertension
VIIP	Visual impairment intracranial pressure
GC	Ground control
SPC	Space flown
HSP-90β	Heat shock protein-90 beta
SPARC	Secreted protein acidic and rich in cysteine
CNS	Central nervous system
OLPs	Oligodendrocyte progenitors
Sim-μG	Simulated microgravity
μG	Microgravity
GC	Ground control
NSCs	Neural stem cells
DM	Dorsomorphin
OSM	Oligodendrocyte specification medium
OSMc	Oligodendrocyte specification medium complete
OSMb	Oligodendrocyte specification medium basal
WM	Welcoming medium
ISS	International space station
STAaRS F-1	Space technology and advanced research system experiment Facility-1
LCC	Long cell cycle
SCC	Short cell cycle
TL	Time lapse
OL	Oligodendroglia
RPMs	Random positioning machines
ER	Endothelial reticulum
